# Growth of MRSA and *Pseudomonas aeruginosa* in a fine-celled foam model containing sessile commensal skin bacteria

**DOI:** 10.1080/08927014.2015.1117607

**Published:** 2016-01-04

**Authors:** Angela Oates, Andrew J. McBain

**Affiliations:** ^a^ Manchester Pharmacy School, The University of Manchester, Manchester, UK

**Keywords:** Colonisation resistance, skin, wound, MRSA, *Pseudomonas aeruginosa*, *Staphylococcus saprophyticus*, *Corynebacterium xerosis*

## Abstract

Sessile cultures of the skin bacteria *Staphylococcus saprophyticus* and *Corynebacterium xerosis* were grown using novel fine-celled foam substrata to test the outcome of challenge by methicillin-resistant *Staphylococcus aureus* or *Pseudomonas aeruginosa* under three growth medium regimens (simulated sweat, simulated serum or simulated sweat substituted with simulated serum during the microbial challenge). *S. saprophyticus* and *C. xerosis* significantly limited MRSA and *P. aeruginosa* immigration respectively, under the simulated sweat and serum medium regimes. Under the substitution medium regime however, MRSA and *P. aeruginosa* integrated into pre-established biofilms to a significantly greater extent, attaining cell densities similar to the axenic controls. The outcome of challenge was influenced by the medium composition and test organism but could not be predicted based on planktonic competition assays or growth dynamics. Interactions between skin and wound isolates could be modelled using the fine-celled foam-based system. This model could be used to further investigate interactions and also in preclinical studies of antimicrobial wound care regimens.

## Introduction

*In vitro* biofilm models have applications ranging from mechanistic studies of biofilm-specific processes to preclinical investigations of antimicrobial products (reviewed by McBain 2009). Various biofilm models have been developed including the constant depth film fermenter (CDFF) in which continuous biofilm cultures of pre-set depth are grown within coupons housed in a rotating turntable (Kinniment et al. 1996; Pratten et al. 1998; McBain et al. 2003a; Ledder et al. 2009). The CDFF has most frequently been used to maintain oral consortia but also to grow mixed populations of wound bacteria (Malic et al. 2009; Hill et al. 2010), for other applications including the growth of aquatic ecosystems (McBain et al. 2004) and to model interactions between *Burkholderia cepacia* and *Pseudomonas aeruginosa* of relevance to cystic fibrosis (Al-Bakri et al. 2004). Whilst the CDFF has proven utility, the rotary action of the turntable and static scraper blades of the CDFF create shear forces that are unlikely to be commonly encountered in wounds. Various other systems, all with inherent strengths and weaknesses, have been adopted to model wound biofilms, including the CDC reactor (Humphreys et al. 2011), porcine dermal explants (Barnea et al. 2010; Yang et al. 2013), microfluidics (Wright et al. 2015), collagen-based systems (Charles et al. 2009) and human skin equivalents generated from primary human keratinocytes (Haisma et al. 2013).

Typically, *in vitro* wound biofilm models utilise immobilised abiotic surfaces in either continuously perfused or batch culture systems. Perfused models including the single (Taylor & Greenman 2010) and multiple Sorbarod devices (Ledder et al. 2009), modified drip slide reactors (Agostinho et al. 2011; Woods et al. 2012), and a specialised perfused biofilm model described by Thorn and Greenman (2009), incorporate porous substrata and have been used to grow biofilms compositionally similar to those occurring in wounds. Static or batch culture systems such as the Lubbock chronic wound model (Dowd et al. 2008; Sun et al. 2008), the modified Lubbock model described by Kucera et al. (2014) and a cellulose disc model described by Hammond et al. (2011) have been used to investigate multispecies wound biofilms and antimicrobial dressings to good effect.

In the current investigation, mono- and binary culture biofilms were grown using water-absorbing thermoset plastic foam (Smithers-Oasis, Washington, UK) marketed for the postharvest maintenance of plants, as substrata. Whilst *in vitro* models have been used to investigate aspects of biofilms formed by bacteria that colonise wounds, particularly in terms of the effect of antimicrobial compounds and dressings (Steffansen & Herping 2008; Sun et al. 2008; Thorn & Greenman 2009; Lipp et al. 2010), interactions between populations of microorganisms associated with intact skin and with dermal wounds have received relatively little research attention. With the broad aim of ascertaining whether growth interactions between bacteria representing skin commensals and wound pathogens could be investigated using a simple *in vitro* system, a fine-celled foam model was used to simulate the challenge of established populations of coagulase-negative staphylococci and *C. xerosis* with the adventitious pathogens methicillin-resistant *Staphylococcus aureus* (MRSA) and *P. aeruginosa*, under growth substrate conditions broadly reflective of intact or wounded skin.

## Materials and methods

### Bacteria

*P. aeruginosa* and *C. xerosis* were isolated from infected wounds; methicillin resistant *S. aureus* NCTC 11939 and *S. saprophyticus* NCTC 7292 were obtained from Public Health England, Southampton, UK.

### Chemicals and media

Unless otherwise stated chemicals used were supplied by Sigma (Poole, UK). Dehydrated bacteriological media were obtained from Oxoid (Basingstoke, UK) and reconstituted according to instructions supplied by the manufacturer.

### Simulated sweat and serum formulations

To simulate and standardise the nutrients typically available to bacteria on intact and wounded skin, growth media broadly reflective of the major constituents of hominine sweat and serum at biologically relevant concentrations were developed (Russell & Wiles 1970; Trengove & Langton 1996). The simulated sweat consisted of 3-(N-morpholino)-propanesulfonic acid (20.9 g l^−1^/100 mM), yeast extract (1 g l^−1^), NaCl (2 g l^−1^), fish oil fatty acid methyl esters (0.65 mg l^−1^) and Tween 80 (0.1 g l^−1^) buffered to pH 6. Simulated serum consisted of MOPS (20.9 g l^−1^/100 mM), NaCl (6.025 g l^−1^), KCl (0.372 g l^−1^), urea (0.54 g l^−1^), creatinine (0.0132 g l^−1^), glucose (0.324 g l^−1^), yeast extract (1 g l^−1^), peptone (3 g l^−1^), MgSO_4_ (0.0168 g l^−1^), haemin (0.005 g l^−1^) and K_3_PO_4_ (0.109 g l^−1^).

### Differential enumeration of bacteria

For mixed cultures of *Staphylococcus saprophyticus* and MRSA, bacteria were enumerated using Wilkins-Chalgren agar supplemented with novobiocin (5 mg l^−1^) or cefoxitin (4 mg l^−1^), respectively; for mixed cultures of *S. saprophyticus* and *P*. *aeruginosa*, Mannitol Salt and *Pseudomonas*-selective agars were used. For cultures of *C. xerosis* combined with MRSA, bacteria were enumerated using Wilkins-Chalgren agar and Wilkins-Chalgren agar supplemented with cefoxitin. For mixed cultures of *C. xerosis* and *P*. *aeruginosa*, Wilkins-Chalgren agar and *Pseudomonas*-selective agar facilitated selective enumeration. Viable counts of *C. xerosis* in combined culture were determined from the total counts derived from Wilkins-Chalgren agar, minus selective counts.

### Bacterial specific growth rates and productivity

Stationary phase cultures of the skin and wound-associated bacterial isolates were then diluted 1:100 in fresh media and 200 μl of the inocula were dispensed into wells of a microtitre plate. Microtitre plates were incubated in an automated plate reader (Titertek Multiskan^®^ MCC 340, Biotek, Swindon, UK) at 37°C for 24 h with optical density readings (600 nm) taken every 20 min. Specific growth rates and delta OD values were determined by the following equations (Inniss & Mayfield 1978) where Χ_1_ and X_2_ are the OD_600_ values on the maximum of the slope of growth curves between times *t*_1_ and *t*_2_.
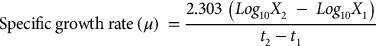





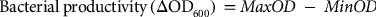



### Relative fitness of combined planktonic cultures

The relative fitness of planktonic cultures of *S. saprophyticus* or *C. xerosis* in pair-wise combination with either MRSA or *P*. *aeruginosa* was assessed using a competitive fitness assay previously described by Lenski et al. (1991) and Rozen et al. (2007). Briefly, stationary phase axenic bacterial cultures maintained in simulated sweat or serum were adjusted to ~8.0 log_10_ CFU ml^−1^ in sterile medium. Pair-wise combinations were mixed in 50:50 ratios and further adjusted to give final inoculum density of ~6.0 log_10_ CFU ml^−1^ of each bacterial isolate. Mixed inocula were serially diluted to verify the initial population densities and were then incubated for 24 h at 37°C. To determine the endpoint densities the mixed inocula were serially diluted and viable counts performed. All pair-wise combinations were tested in triplicate.

### Use of a fine-celled foam multi-well plate wound model to investigate community integration and colonisation resistance

**Figure 1.**
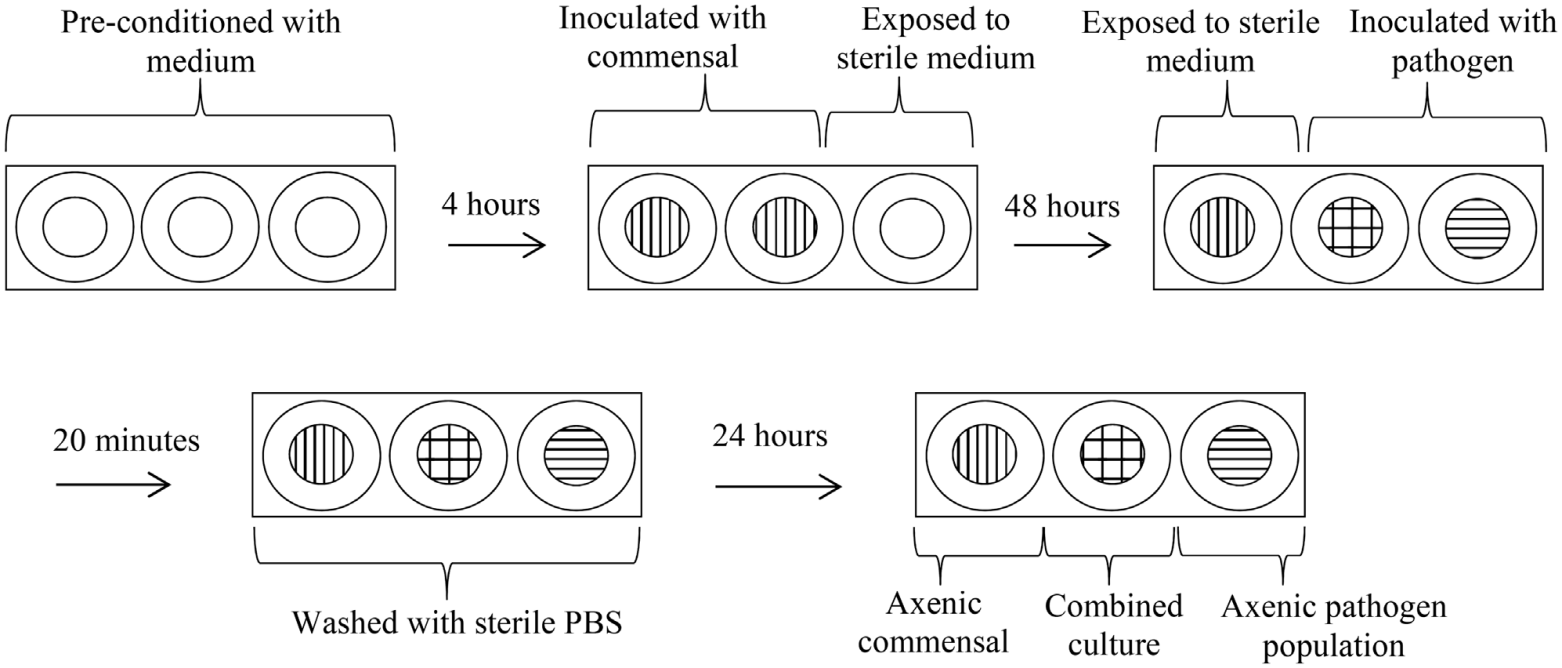
Schematic diagram of the biofilm model experiments.

To investigate interactions between established and introduced bacteria, a fine-celled foam (FCF) multi-well model was used. A fine celled thermoset phenolic plastic foam (Smithers-Oasis) was selected as a model substratum. This foam consists of a cellular solid structure with interconnecting pores, which drives medium uptake by capillary action. It is structurally stable when saturated and has excellent wet heat stability facilitating sterilisation by autoclaving. Sterile FCF portions were cut to column size (height 1 cm × 1 cm diameter) and placed within the wells of a sterile 24 multi-well culture plate (Sigma). An overview of the inoculation procedure and incubation steps is shown in Figure 1. Briefly FCF substrata were pre-conditioned in simulated media for 4 h and then either inoculated with 1.0 ml stationary phase cultures of *S. saprophyticus* or *C. xerosis* (adjusted to ~7.0 log_10_ CFU ml^−1^) or left as uninoculated controls. The FCF substrata were then incubated aerobically at 37°C*.* After 48 h incubation, inoculated and uninoculated substrata were exposed to 1 ml of ~7.0 log_10_ CFU ml^−1^ of MRSA or *P. aeruginosa*, with the remaining inoculated substrata exposed to 1 ml of sterile medium for 20 min. All FCF substrata were then immersed in sterile PBS (pH 7.0, 0.01 M), and returned to their original wells containing fresh sterile medium. After further incubation for 24 h, the FCF substrata were aseptically removed and placed into plastic Universal bottles (Scientific Laboratory Supplies, Nottingham, UK) containing 9 ml of half-strength thiogylcolate broth and 5 mm sterile glass beads (*n* = 5) (Merck, Darmstadt, Germany). To ensure the disintegration of the substrata, effective extraction of bacterial cells, and uniform distribution of cells throughout the diluent, the Universal bottles and contents were mixed at 800 rpm on a reciprocal flask shaker (Griffin & George, Loughborough, UK) for 2 min. Extracted cells were then serially diluted in half-strength thiogylcolate broth and plated on variously selective agars, as outlined above (McBain et al. 2003b; Matejka et al. 2012). All substrata were maintained under fed-batch conditions with spent medium removed and replaced with fresh medium every 24 h. All experiments were undertaken in triplicate and repeated under three growth medium regimens: (1) simulated sweat; (2) simulated serum; or (3) simulated sweat with medium switched to simulated serum during the microbial challenge. Statistical significance was determined using independent *t*-test and Mann–Whitney test to determine significant difference.

## Results

### Bacterial specific growth rates and productivity in formulated media

Both simulated sweat and serum sustained the growth of the test bacteria although growth rates and productivity for both MRSA (0.201 and 0.838 h^−1^) and *P. aeruginosa* (0.136 and 0.833 h^−1^) were markedly higher than for the cutaneous organisms *C. xerosis* (0.098 and 0.610 h^−1^) and *S. saprophyticus* (0.106 and 0.470 h^−1^) in simulated sweat and serum, respectively (Table 1).

**Table 1.** Specific growth rate and delta OD values of organisms grown in formulated simulated sweat.

	Simulated sweat		Simulated serum	
	μ	ΔOD	μ	ΔOD
*P. aeruginosa*	0.136	0.206	0.833	0.392
MRSA	0.201	0.249	0.838	0.348
*S. saprophyticus*	0.106	0.153	0.470	0.218
*C. xerosis*	0.098	0.130	0.610	0.368

μ, specific growth rate; ΔOD, bacterial productivity assessed by change in optical density.

### Relative fitness of combined planktonic cultures

When grown together in binary planktonic culture, MRSA and *P. aeruginosa* were significantly fitter than *C. xerosis* and *S. saprophyticus* in both the simulated serum and sweat (Table 2).

**Table 2.** Relative fitness of selected organisms when combined in co-culture.

	Simulated sweat				Simulated serum			
	*C. xerosis*		*S. saprophyticus*		*C. xerosis*		*S. saprophyticus*	
	*P*	*C*	*P*	*C*	*P*	*C*	*P*	*C*
MRSA	1.29 (0.04)*	0.77 (0.03)*	1.72 (0.2)*	0.58 (0.06)*	1.20 (0.09)*	0.84 (0.06)*	1.27 (0.07)*	0.79 (0.05)*
*P. aeruginosa*	1.33 (0.07)*	0.75 (0.04)*	1.20 (0.06)*	0.83 (0.04)*	1.85 (0.19)*	0.55 (0.06)*	1.81 (0.15)*	0.51 (0.11)*

*P*, relative fitness values of pathogenic organisms to the commensal organisms; *C*, relative fitness values of the commensal organisms to the pathogenic organisms. Standard deviations are given in the parenthesis. Asterisks indicate significant differences (*p* < 0.05). Values > 1 indicate advantageous growth of the organism in co-culture.

### Bacterial integration and colonisation resistance

**Figure 2.**
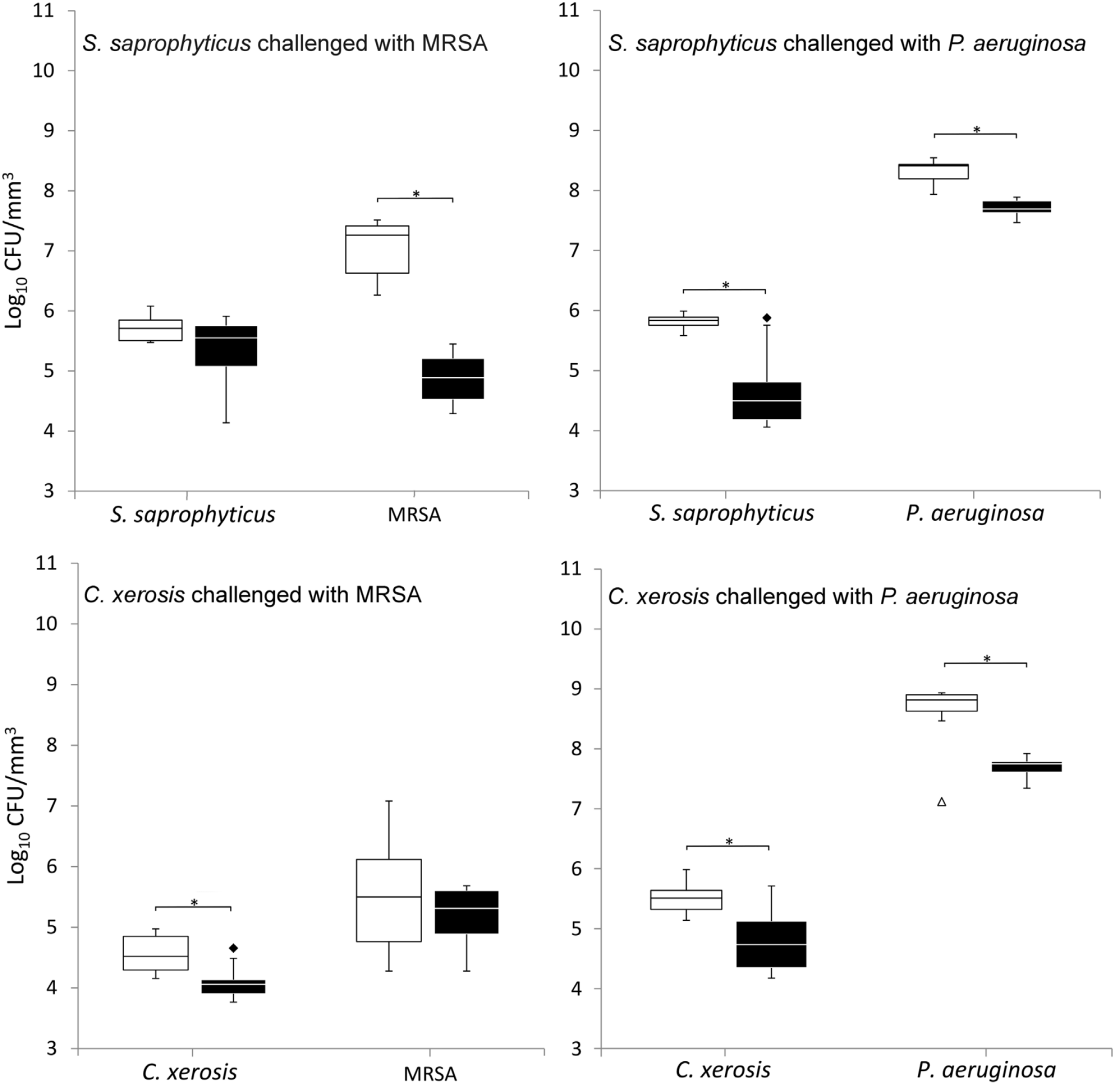
Viable counts of axenic and combined cultures of pre-established communities of *S. saprophyticus* and *C. xerosis* when exposed to the transient pathogenic bacteria MRSA and *P. aeruginosa* in simulated sweat. Data are means of three separate experiments. Asterisks indicate significant differences (*p* < 0.05). White boxes, axenic culture; black boxes, binary culture; minimum and maximum outliers are also indicated.

**Figure 3.**
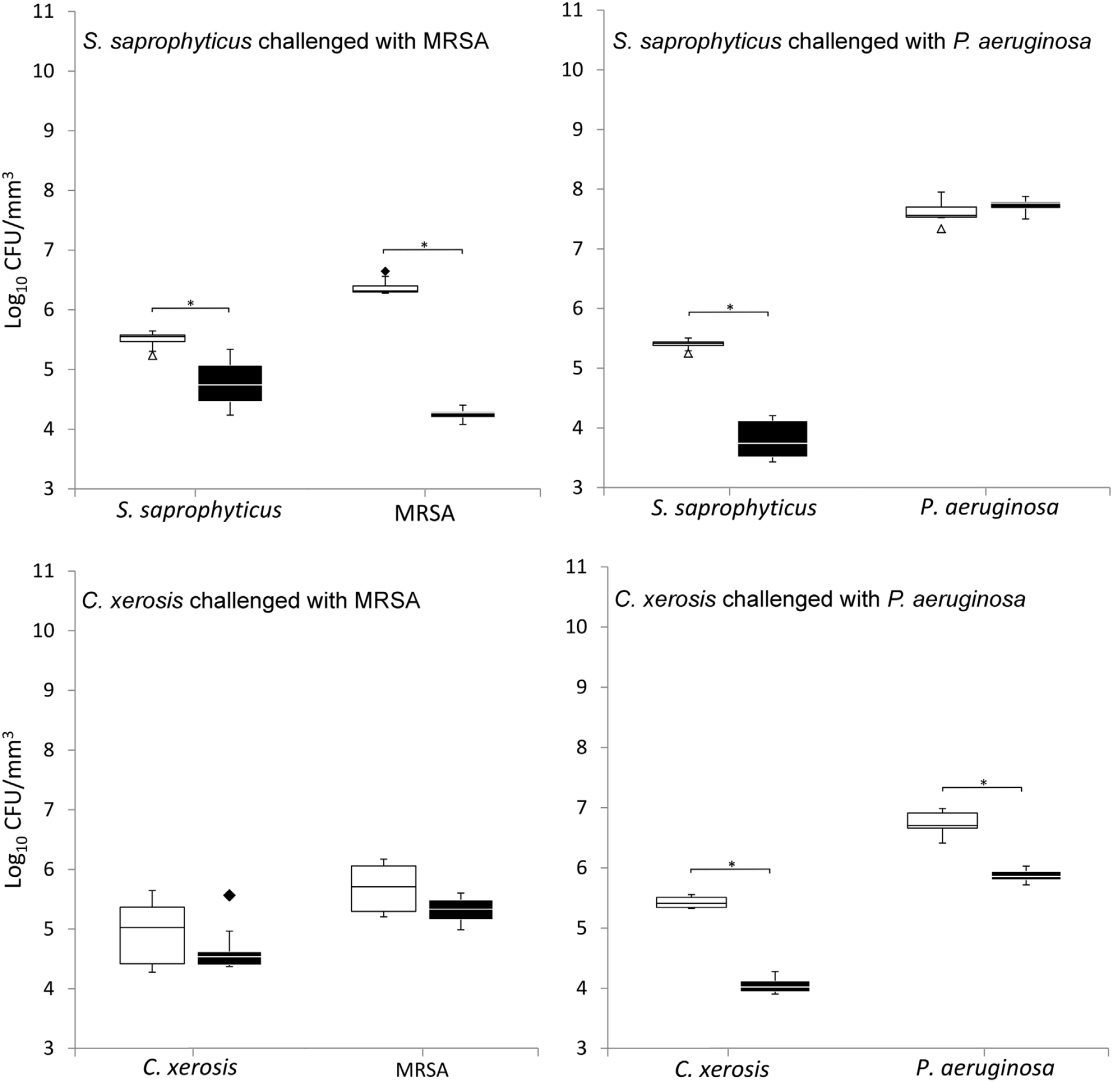
Viable counts of axenic and combined cultures of pre-established communities of *S. saprophyticus* and *C. xerosis* when exposed to the transient pathogenic bacteria MRSA and *P. aeruginosa* in simulated serum. See legend to Figure 2.

**Figure 4.**
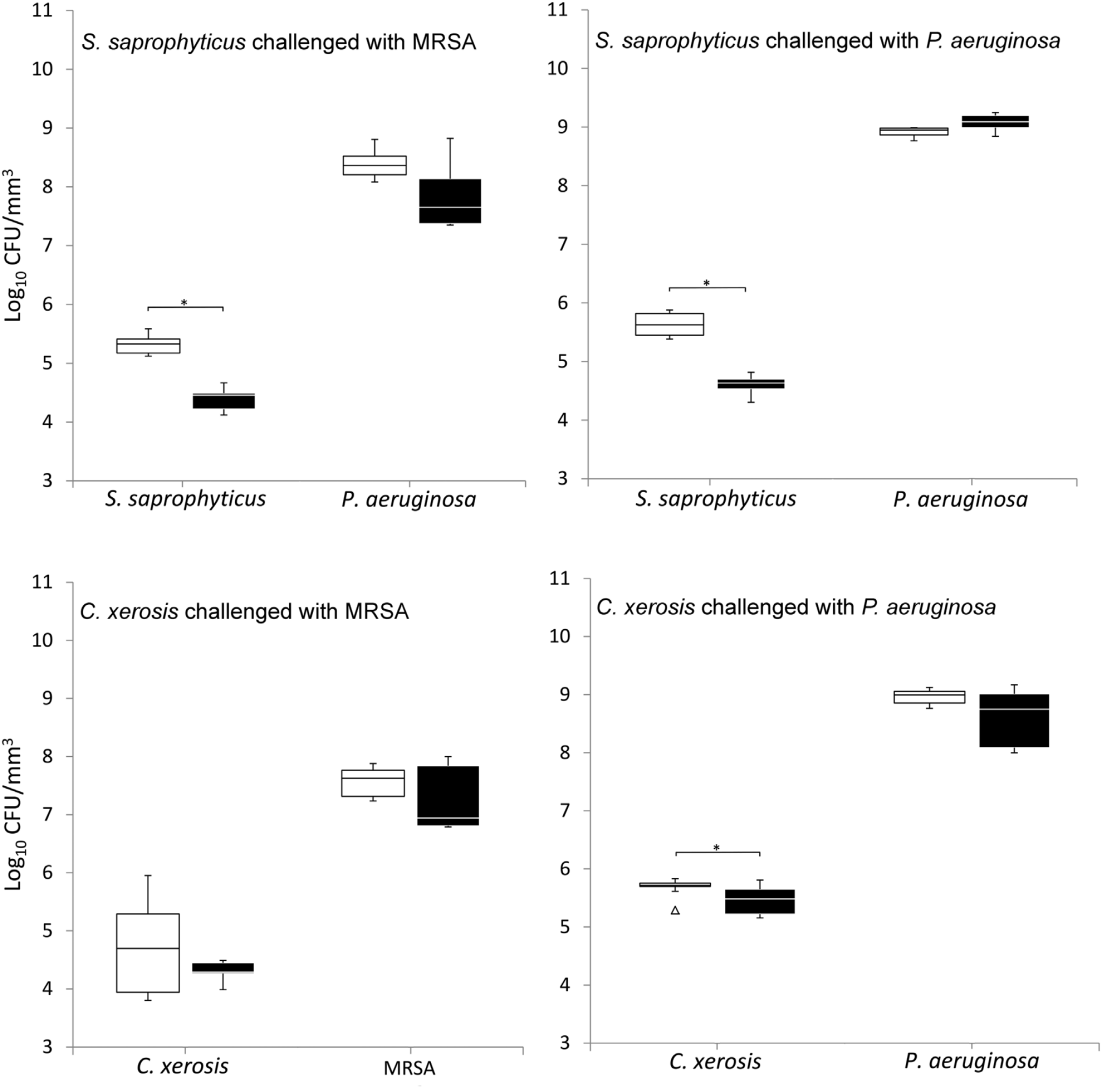
Viable counts of axenic and combined cultures of pre-established communities of *S. saprophyticus* and *C. xerosis* when exposed to the transient pathogenic bacteria MRSA and *P. aeruginosa* in simulated sweat with medium substituted with simulated serum during microbial challenge. See legend to Figure 2.

When grown in simulated sweat or serum and compared to non-colonised controls, prior colonisation of *S. saprophyticus* and *C. xerosis* resulted in significantly reduced integration of MRSA and *P. aeruginosa* on FCF substrata, as shown in Figures 2 and 3 (*p* < 0.05) in five of eight cases, with the exception of *S. saprophyticus* when challenged with *P. aeruginosa* under simulated serum and *C. xerosis* when challenged with MRSA under simulated serum and sweat. Significant displacement of the pre-colonised organisms also occurred (p < 0.05) with viable counts of *C. xerosis* significantly lower (p < 0.05) than for axenic controls when challenged with MRSA under simulated sweat feeding and when challenged with *P. aeruginosa* under both simulated sweat and serum (Figures 2 and 3). Significantly lower densities of *S. saprophyticus* were detected when challenged with *P. aeruginosa* in simulated sweat (Figure 2) and when challenged by either pathogen under simulated serum (Figure 3). Under the substitution nutrient regime, both MRSA and *P. aeruginosa* successfully integrated into established cultures of *S. saprophyticus* and *C. xerosis*, producing population densities that were not significantly different from their axenic controls (Figure 4). To check for the presence of sessile bacteria and biofilms within the FCF material, selected samples were visualised using environmental scanning electron microscopy. According to this, putative attached microcolonies were present on the surfaces of inoculated FCF substrata that were absent on the control material (images not shown).

## Discussion

The presence (Malic et al. 2009; Oates et al. 2014) and purported role biofilms play in wounds (Bjarnsholt et al. 2008; James et al. 2008) has been the focus of considerable attention in wound healing research and there is substantial commercial interest in developing dressings and combinations of antimicrobials with enhanced anti-biofilm activity (Kostenko et al. 2010; Kucera et al. 2014). Various *in vitro* models have been used to study wound biofilms (Sun et al. 2008; Thorn & Greenman 2009; Dalton et al. 2011; Kucera et al. 2014) and to facilitate the preclinical assessment of potential treatment outcomes (Kostenko et al. 2010; Agostinho et al. 2011; Woods et al. 2012). Interactions between pathogens associated with wounds and sessile populations of ‘commensal’ organisms have however received relatively little research attention. As with other microbial communities associated with the human body where microbially mediated colonisation resistance is likely to be an important protective process, it is believed that the skin microbiota, although of comparatively low density (normally ranging between 10^2^ CFU cm^−2^ and 10^7^ CFU cm^−2^; Fredricks 2001), can inhibit the attachment and proliferation (Axelsson & Mahida 2000) of adventitious pathogens *via* competition for attachment sites, nutrients and the production of inhibitory metabolites such as lactic acid and antimicrobial molecules such as bacteriocins (McAuliffe et al. 2001; Varella Coelho et al. 2007).

With the aim of using a biofilm model based on a fine-celled foam substratum to investigate the outcome of binary bacterial interactions of the type that may occur on the skin in health and during wounding, pair-wise interactions between two organisms typically isolated from the epidermis (*S. saprophyticus* and *C. xerosis*) or associated with wound infections (MRSA and *P. aeruginosa*) were studied under growth substrate conditions broadly reflective of intact or wounded skin using a biofilm model based on fine-celled foam. The organisms were selected based on their common isolation from either the epidermis or wounds, combined with their antibiograms and colony morphology on selective agar plates, which facilitated selective growth of individual organisms when grown in defined consortia.

The capacity of the test bacteria to grow in the formulated media was evaluated and all grew in both simulated serum and sweat although considerably higher growth rates and bacterial productivity occurred for the wound pathogens. This was reflected in the outcome of pair-wise growth interactions in relative fitness assays in which ratios of the growth rates of each organism in co-culture were determined. When planktonic cultures of MRSA or *P. aeruginosa* were combined with either *S. saprophyticus* or *C. xerosis*, significant advantageous growth for both pathogens occurred. As no antagonistic activity between these organisms was detected using deffered or direct antagonism assays (data not shown), this suggests that the competitiveness of the pathogenic species was most likely due to relative growth rates rather than antagonism. The utility of a fine-celled foam model for investigations of bacterial community integration, displacement or colonisation resistance and the influence of variable nutritional conditions on these factors was then assessed.

In the majority of previous reports into the use of *in vitro* models to simulate aspects of the bacteriology of human skin and wounds, a range of model systems have been used mainly to assess the attachment and biofilm formation by bacteria associated with wounds (Sun et al. 2008; Malic et al. 2009; Thorn & Greenman 2009), their responses to antibiotics (Gander et al. 2002) or to test topical antimicrobials (Dowd et al. 2009; Lipp et al. 2010). The current investigation differs from some previous reports in that it assesses the use of a biofilm model system to investigate interactions between bacteria. A novel substratum material was used in 24-well plate system, thus facilitating biological replication, and nutrient conditions were varied in order to broadly simulate conditions associated with healthy skin (simulated sweat medium) and chronic wounds (simulated serum) and through the use of varied nutrient conditions to represent wounding (medium substitution) intended to simulate changes in nutrient availability likely to occur during wounding, from the nutrient-limited environment of the skin to the more nutritious environment of a wound. During the validation phase of the study, inoculated FCF substrata were imaged using environmental scanning electron microscopy to determine whether biofilms were present within the substrata. Structures resembling attached biofilm microcolonies were observed on inoculated substrata, but not on uninoculated controls.

Under the simulated sweat and serum regimes, limited integration of the wound isolates into established populations of either *S. saprophyticus* or *C. xerosis* occurred, an outcome which could not be predicted based on planktonic pair-wise competition assays or planktonic growth dynamics, where both MRSA and *P. aeruginosa* consistently outcompeted *S. saprophyticus* and *C. xerosis* when grown in simulated sweat or serum. Interestingly, under the medium substitution regime, significantly greater integration of the pathogens into pre-established commensal populations occurred, resulting in populations of the pathogens that reached similar densities to the control substrata. This may result from detachment of the pre-established bacterial communities in response to rapidly increased nutrient availability as the growth medium was substituted, possibly allowing greater adherence of the pathogenic species. This is supported by the observation that the resistance to colonisation was greater when *S. saprophyticus* and *C. xerosis* were continually grown in simulated serum.

The intention of this investigation was not to directly simulate the epidermal and wound environment, but rather, to assess the potential of a biofilm model utilising a novel fine-celled foam substratum for use in investigations into physiological and ecological interactions between specific groups of wound and skin isolates, under controlled conditions. Colonisation resistance, which varied depending on the medium environment and the commensal and pathogenic bacteria, could be broadly quantified in the FCF model. The model could be further applied to investigate bacteriotherapy for wounds and to model the effects of selective antimicrobial treatments on the bacteriological composition of biofilms grown using wound isolates.
